# Kin Discrimination Increases with Genetic Distance in a Social Amoeba

**DOI:** 10.1371/journal.pbio.0060287

**Published:** 2008-11-25

**Authors:** Elizabeth A Ostrowski, Mariko Katoh, Gad Shaulsky, David C Queller, Joan E Strassmann

**Affiliations:** 1 Department of Ecology and Evolutionary Biology, Rice University, Houston, Texas, United States of America; 2 Department of Molecular and Human Genetics, Baylor College of Medicine, Houston, Texas, United States of America; University of Edinburgh, United Kingdom

## Abstract

In the social amoeba Dictyostelium discoideum, thousands of cells aggregate upon starvation to form a multicellular fruiting body, and approximately 20% of them die to form a stalk that benefits the others. The aggregative nature of multicellular development makes the cells vulnerable to exploitation by cheaters, and the potential for cheating is indeed high. Cells might avoid being victimized if they can discriminate among individuals and avoid those that are genetically different. We tested how widely social amoebae cooperate by mixing isolates from different localities that cover most of their natural range. We show here that different isolates partially exclude one another during aggregation, and there is a positive relationship between the extent of this exclusion and the genetic distance between strains. Our findings demonstrate that D. discoideum cells co-aggregate more with genetically similar than dissimilar individuals, suggesting the existence of a mechanism that discerns the degree of genetic similarity between individuals in this social microorganism.

## Author Summary

In social amoebae such as Dictyostelium discoideum, cells aggregate to form a multicellular slug that migrates and then forms a fruiting body, which contains live spores (which go on to make new amoebae) and dead stalk cells. Unlike animals where all the cells descend from one fertilized egg, social amoeba fruiting bodies can contain cells with different genotypes. This potential for chimerism creates a conceptual problem in that “cheater” cells could arise that preferentially become reproductive spores and force the victims to become stalk cells and die. One way that amoebae could avoid being cheated is if they recognize and preferentially aggregate with genetically similar cells while avoiding genetically distant cells—a process called kin discrimination. We tested whether cells of D. discoideum could discriminate in this way. We mixed cells from genetically distinct strains and found that they segregate during multicellular development. The degree of segregation increases in a graded fashion with the genetic distance between strains. Our results demonstrate the existence of kin discrimination in D. discoideum, an ability that is likely to reduce the potential for cheating and ensure that the death of the stalk cells provides a fitness advantage to related individuals.

## Abstract 3

Genetically based discrimination in social amoebae may help these cells to avoid cheaters that take advantage of their altruistic behavior.

## Introduction

The ability to recognize and preferentially interact with kin can favor the evolution of altruistic or cooperative traits [1,2]. Microorganisms exhibit complex social behaviors [3–6], but little is known about the genetic and geographic scale of their cooperation [3]. Social traits, in particular, may be prone to the emergence of incompatibilities: selection to avoid potential costs of cooperation, including cheating, may drive rapid evolution at discrimination or other loci and limit cooperation to closely related strains.

The social amoeba D. discoideum (formerly known as the cellular slime mold) offers a unique opportunity to examine the relationship between genetic distance and discrimination in a cooperative microbe. It is haploid, and its genome contains numerous microsatellite loci, which permit quantitative estimation of genetic differences between individuals. It has a geographically restricted range and is found primarily in forest soils of eastern North America and East Asia [7]. Upon starvation, unicellular amoebae assemble in groups of approximately 10^4^–10^5^ cells to form a multicellular aggregate. The aggregate can migrate toward light and heat and eventually develop into a fruiting body composed of a ball of spores held aloft by a rigid cellular stalk. Approximately 70–80% of the cells in the initial aggregate will form spores, whereas 20–30% of the cells will die and form the stalk. Stalk formation is considered to be altruistic, because stalk cells die to benefit the spores by lifting them above the ground, which may increase their chances of dispersal and protect them from hazards in the soil while they sporulate [8–11].

Aggregation in D. discoideum can occur between amoebae that are genetically different, and so evolutionary theory predicts selection for cheaters—genotypes that gain the benefit of the stalk while failing to contribute their fair share to its production [12–15]. Indeed, studies of natural isolates have shown that genetically distinct strains of D. discoideum can form chimeras in the laboratory that can differ in their allocation to the prespore versus prestalk regions of the slug [8]. Genetic screens to examine cheating behavior in the laboratory strain have also revealed numerous genes that, when disrupted, lead to that mutant's overrepresentation in the spores [16].

The demonstrated ubiquity and ease of social cheating in D. discoideum pose a conundrum—what maintains the victims in nature? One possibility is that cheaters have lower fitness than cooperators when not in chimeras. If this is the case, then the fitness advantage gained by cheaters might be reduced or eliminated by mechanisms that lead to the separation of cheaters and cooperators into distinct fruiting bodies [14,17,18]. There are two explanations for how this separation might occur. One possibility is that cheaters and victims rarely interact, because population structure passively leads to the formation of primarily clonal fruiting bodies. Another possibility is that strains segregate from one another before or during multicellular development, a form of kin discrimination. Kin discrimination differs from kin recognition in that the latter term refers to cognitive processes, whereas kin discrimination describes observable behavioral patterns [19–22]. Evidence for kin discrimination is provided by a study in a different species (D. purpureum), which showed that cells segregated from non-identical cells during multicellular development, although no cheating was observed [23]. In D. discoideum, however, evidence for discrimination is indirect only: genetically distinct clones are found in close proximity in the soil [24], but fruiting bodies are often dominated by a single clone, at least on the rich substrate of deer feces where the majority of wild fruiting bodies have been found [17].

D. discoideum is a genetically tractable model system, and so understanding whether it is capable of detecting and restricting cooperation in accordance with genetic distance is an important step toward identifying the genetic basis of the underlying mechanisms. For example, studies of *csaA* mutants of D. discoideum, which lack the cell–cell adhesion molecule gp80, have shown that *csaA^–^* cells tend to be lost from chimeric aggregates with wild-type cells on natural substrates, suggesting that differences in cell adhesion among strains could facilitate discrimination [25,26]. However, segregation between wild isolates of D. discoideum has not been reported, making the relevance of this finding unclear.

We examined several patterns of discrimination in D. discoideum. First, we tested directly whether genetically different isolates are capable of segregating from one another during multicellular development. Second, we determined the degree to which the genetic distance between strains influences the extent of this exclusion. Finally, we examined the phenotypic basis of segregation among different mixes in light of different possible explanations for sorting based on previous work [27,28].

## Results

To examine the relationship between the genetic similarity of strains and the amount of segregation they exhibit during the formation of fruiting bodies, we performed pairwise mixes of a reference strain and a panel of natural isolates (Table S1). To estimate the genetic distances between strains, we genotyped them at 12 polymorphic microsatellite loci, which were dispersed throughout the genome. We calculated the standardized Euclidean distance between strains based on their microsatellite allele sizes and used it as an estimate of genetic divergence, and thus as a proxy for the probability that strains share alleles (Table S2). Genetic distance is thus similar to relatedness in that both measures are estimates of identity by descent, although they differ formally, since the latter is expressed relative to allele frequencies in a reference population [29,30]. More important, because genetic distance takes into account not just allelic identity but distance between alleles, it provides greater resolution than relatedness measures based on shared alleles for divergent strains sampled from different geographic locations.

We first mixed the laboratory strain AX4-GFP (labeled by transformation with the gene for green fluorescent protein) with each of 14 natural isolates, the strain from which it was derived (natural isolate NC4), and unlabeled AX4 (control). For each mix, we combined labeled and unlabeled amoebae in equal proportions, deposited the mixture on damp nitrocellulose filters, and allowed them to aggregate and form fruiting bodies. We sampled ten fruiting bodies from each mix and determined the ratio of fluorescent to nonfluorescent spores in each one. We used the average variance in this proportion across fruiting bodies, based on a minimum of three temporally independent replicates, as an estimate of the degree of segregation for a given pair of strains.

**Figure 1:**
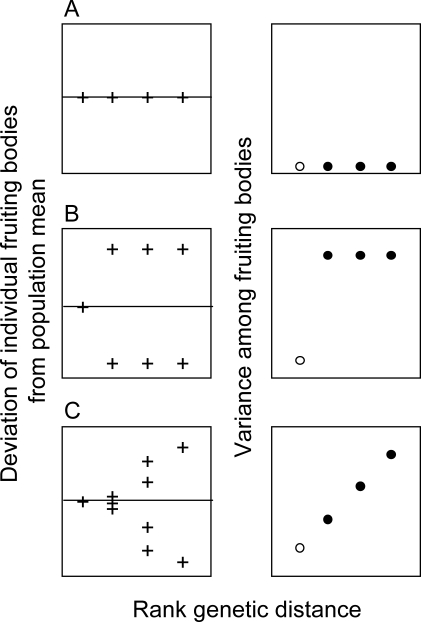
Hypothetical Patterns of Discrimination (Left panel) Deviation of individual fruiting bodies from the mean of the population. Each symbol (+) represents an individual fruiting body, and mixes are plotted in order of increasing genetic distance. (Right panel) Variance among fruiting bodies plotted as a function of genetic distance. Open circles represent the control mix between genetically identical labeled and unlabeled cells. Full circles represent mixes between genetically distinct cells. We consider three hypotheses: (A) No discrimination. The left panel shows each fruiting body contains similar proportions of the two clones. The right panel shows the resulting variance among fruiting bodies is low and there is no difference between self-mixes (open circle) and non-self mixes (full circles). (B) Exclusive self–nonself discrimination. The left panel shows the labeled strain mixes well with genetically identical cells but poorly with other clones. The right panel shows there is a difference between the variance of the self mix and the nonself mixes, but no difference among the nonself mixes. (C) Discrimination according to genetic similarity. The left panel shows mixes of genetically identical cells produce well-mixed fruiting bodies, but segregation into distinct fruiting bodies is observed as the genetic distance between clones increases. The right panel shows increasing variance is proportional to the genetic distance between strains.

The mixing experiment could have several outcomes (Figure 1). In the absence of any discrimination, all fruiting bodies should show identical proportions of the two clones, resulting in low variance in that measure and no differences between mixes of isolates at different genetic distances (Figure 1A). Under exclusive self–nonself discrimination, individuals would be expected to cooperate and form fruiting bodies with genetically identical cells but segregate from all other strains, resulting in a strongly binary response (Figure 1B). Alternatively, if the degree of discrimination depends on the genetic similarity between the strains, we expect to see a graded relationship between genetic distance and the degree of sorting (Figure 1C).

**Figure 2:**
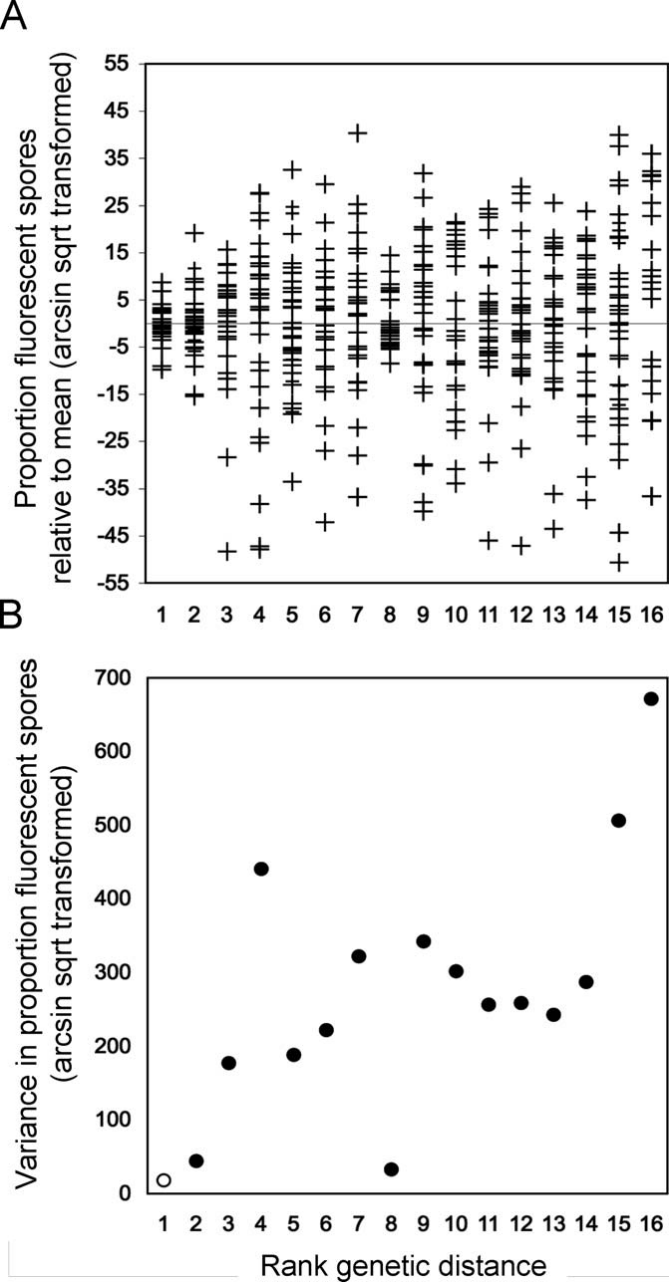
Segregation Increases with Genetic Distance in Mixed Fruiting Bodies Reference cells (AX4-GFP) were mixed in equal proportions with test cells of various genetic distances, and the mixes were allowed to form fruiting bodies. The numbers of GFP-positive and negative spores were determined in ten individual fruiting bodies for each of three or four independent mixes. (A) Combined data from replicate mixes showing the proportion of GFP-positive spores in each fruiting body (+), centered around the mean and plotted as a function of the rank genetic distance from the reference strain AX4. (B) The average variance in the proportion of GFP-positive spores for each of 16 strains, plotted as above, based on three or four independent mix experiments for each strain pair. The correlation between the variance and the genetic distance was positive and statistically significant (Spearman's correlation ρ = 0.631, *n* = 16, *p* = 0.009), indicating greater segregation with increased genetic distance. Data for strains QS33 and QS32 are plotted separately (rank genetic distances 11 and 12, respectively) but were assigned tied ranks for the purposes of calculating the Spearman rank correlation coefficient.

The results of the mixing experiments support the third model (Figure 2). When AX4-GFP was mixed with either unlabelled AX4 (control) or with the parental wild isolate NC4 (rank genetic distance 1 and 2, respectively), the proportion of GFP-positive spores was similar between different fruiting bodies (Figure. 2A) and the variance was low (Figure 2B), indicating low sorting. However, mixes of AX4-GFP with isolates of increasing genetic distance resulted in greater segregation, reflected in the higher variance, and mixes of the most genetically distant strains resulted in fruiting bodies of two classes, indicating that the strains segregated from one another (Figure 2). We observed a highly significant correlation between the genetic distance and the variance (Pearson correlation coefficient: *r* = 0.773, *n* = 16, two-tailed *p* <0.0001), indicating that segregation increased in proportion to the genetic distance between strains. Because the genetic distances were non-normally distributed, we also performed a nonparametric correlation, which was also highly significant (Spearman rank correlation: ρ = 0.631, *n* = 16, two-tailed *p* = 0.009). Finally, despite limited resolution to discriminate between the more distantly related strains, analyses in which genetic distance was estimated based on the number of shared alleles rather than allele size differences produced similarly significant results (Spearman rank correlation: ρ = 0.798, *n* = 16, *p* = 0.0002).

**Figure 3:**
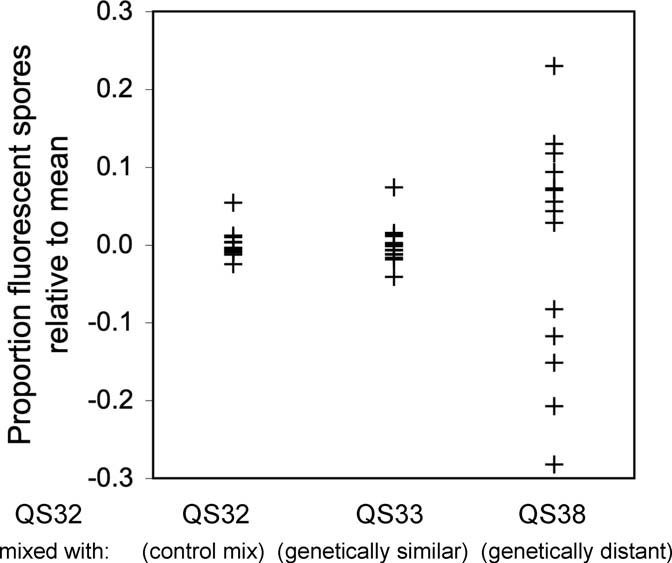
The Property of Segregation Is Transitive and Robust to Strain Choice Labeled reference cells (QS32) were mixed at equal proportions with unlabeled test cells: QS32 (genetically identical), QS33 (identical by 12 genetic markers but isolated from a different geographic location), and QS38 (identical at one genetic marker). The mixed cells were allowed to form fruiting bodies and the numbers of fluorescent and nonfluorescent spores were determined in at least ten fruiting bodies. The proportion of fluorescent spores in each fruiting body (+) is plotted as a function of the rank genetic distance from the reference strain QS32.

To test the generality of the result, we repeated our experiments with a different combination of strains. We chose two natural isolates (QS32 and QS33), which mixed poorly with AX4-GFP but were closely related to one another (identical at all microsatellite loci we examined), and a third strain (QS38), which was equally dissimilar to both (Table S1). If the degree of discrimination can be predicted on the basis of genetic similarity, then the genetically similar strains QS32 and QS33 should mix well with one another and segregate from the genetically distant strain QS38. To test this prediction, we labeled the strain QS32 with a vital fluorescent dye and developed it in pairwise mixtures with the other two strains and with unlabeled QS32 cells as a control. We observed little segregation in the control mix and in the mix of the genetically similar strains QS32 and QS33 (Figure 3). By contrast, mixing QS32 with the genetically distant strain QS38 resulted in fruiting bodies with more variable proportions of labeled spores, indicating stronger segregation. These results are consistent with the studies performed with the labeled laboratory strain, suggesting that the property of genetically related segregation is transitive [31] and robust to strain choice.

**Figure 4:**
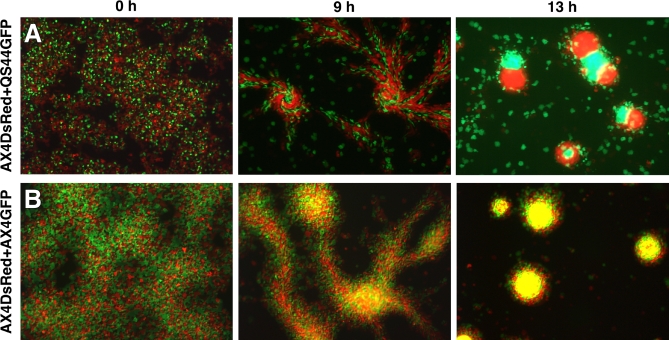
Sorting of Strains during Multicellular Development Cells expressing either GFP or DsRed were mixed at equal proportions and allowed to develop on agar plates. Pictures were taken at the indicated developmental time points and the merged image of the two fluorophores is shown. (A) A mix of the genetically dissimilar strains AX4-DsRed and QS44-GFP shows increased segregation with time. (B) A mix of the genetically identical strains AX4-DsRed and AX4-GFP shows no segregation.

Segregation could result from differential aggregation or from post-aggregative segregation. To distinguish between these possibilities, we transformed one of the natural isolates with a GFP-expression vector (QS44-GFP) and mixed it with a genetically dissimilar strain, labeled with a DsRed expression vector (AX4-DsRed, Figure 4A). As a control, we also mixed AX4-GFP cells with AX4-DsRed cells (Figure 4B). In both the experiment and control mixes, the labeled and unlabeled cells were well mixed when initially plated on agar to induce development (Figure 4A and 4B, 0 h). As development progressed, both mixes initiated aggregation and all the cells moved toward the same aggregation centers regardless of their genetic similarity (Figure 4A and 4B, 9 h). However, clusters of differentially labeled cells became increasingly evident in mixes of the genetically dissimilar strains (Figure 4A, 9 h) whereas the genetically identical cells remained intermixed (Figure 4B, 9 h). Segregation of the genetically dissimilar strains continued throughout the aggregation stage, at which point there was partial separation of labeled and unlabeled cells into different aggregates, as well as segregation within aggregates (Figure 4A, 13 h). Thus, genetically different strains segregate, but they do so imperfectly. The control mixes showed no segregation at that stage or at any later time (Figure 4B, 13 h, and unpublished data). We observed similar segregation in mixes of AX4 with the genetically different isolates QS32 and QS38 (unpublished data). The post-aggregative nature of segregation suggests that the sorting does not result from differences in developmental timing or the use of different chemoattractants, which are known to reduce interspecific chimerism [27].

## Discussion

Our findings demonstrate that social amoebae discriminate between genetically similar and dissimilar cells in a graded manner, mixing more with the former and segregating from the latter during multicellular development. The similarity of the segregation patterns among a number of different isolates suggests a common underlying mechanism and is consistent with differences that could arise from differential cell adhesion [28]. For example, cell-cell adhesion is required for cell streaming, and the spatial segregation of the prestalk and prespore cells, which also occurs in the mound following aggregation, is attributed to differences in the relative adhesiveness of these different cell types [26,32–35].

We propose two evolutionary explanations for these discrimination patterns, which are not mutually exclusive [31,36,37]. One possibility is that genetic drift or adaptation to different components of the environment drives genetic divergence at the loci that cause discrimination, ultimately resulting in a diminished ability to form chimeric fruiting bodies, a process analogous to allopatric speciation [38]. Alternatively, divergence could result from selection to avoid the costs of chimerism, including cheating. Previous studies in D. discoideum have shown that cheaters are abundant in nature [8,39] and that its genome contains numerous genes that confer cheating behavior when mutated [16]. Knowledge of the distribution of genetic variation in nature may help to determine how often foreign individuals encounter one another and the importance of selection in driving the discrimination patterns we show here [31]. Nevertheless, our findings provide a possible explanation for the high levels of clonality observed in fruiting bodies in natural populations [17], as well as the co-existence of cheaters and victims in close proximity in nature [24].

Although we observed a strong correlation between genetic distance and the degree of segregation, most fruiting bodies retained some representation of both clones, indicating some ability to coaggregate and form multicellular structures that extends across nearly the entire species range. Moreover, the demonstrated ability of these strains to segregate, but their failure to do so completely, suggests that an important component of elucidating the role of selection on discrimination ability will involve quantifying not only the costs to forming chimeras, such as cheating, but also the benefits, including increased aggregate size [31,40]. Our results also differ from most other examples of discriminatory behaviors in microbes, such as toxin production in bacteria [41] and vegetative incompatibility in fungi [42]. In those cases, discrimination behaviors show strong self–nonself recognition, and the discrimination phenotypes, which often involve cell death, are strongly binary.

Interestingly, social incompatibilities also occur in the soil bacterium Myxococcus xanthus, which also forms multicellular fruiting structures in response to starvation. In that system, strong antagonism is observed between geographically distinct isolates, with mixes causing reductions in sporulation or even population extinction [43]. By contrast, we observe little evidence of antagonism but increasing avoidance of sociality with genetically dissimilar strains, a behavior that should limit the fitness advantages afforded by cheating and help to explain the maintenance of altruism in this species. More generally, the differences between M. xanthus and D. discoideum in their response to foreign individuals illustrate that different microbes, despite broad similarities in their social life history traits, may find different solutions to the problem of ensuring cooperation [44,45].

## Materials and Methods

### Strains and culture conditions.

In mixes with AX4-GFP, we grew all competitor strains, including AX4, on SM-agar plates (per liter: 10 g glucose, 10 g Bacto Peptone (Oxoid), 1 g yeast extract (Oxoid), 1g MgSO_4_, 1.9 g KH_2_PO_4_, 0.6 g K_2_HPO_4_, 20 g agar) in association with Klebsiella pneumoniae at room temperature. We grew the reference strain AX4-GFP axenically in HL5 medium supplemented with 5 μg/ml G418 with shaking at 22 °C to maintain GFP expression [46].

### Mixing experiments.

We harvested each strain during the mid-exponential phase of growth, washed the cells twice with cold KK2 buffer (14.0 mM K_2_HPO_4_ and 3.4 mM K_2_HPO_4_, pH = 6.4), and resuspended them at a density of 6 × 10^7^ cells/ml in KK2 buffer. For each mix, we combined the two strains in equal proportions and deposited an aliquot of 1.5 × 10^7^ cells on a nitrocellulose filter at a density of 3.5 × 10^6^ cells/cm^2^. As a control, we also plated each strain individually at the same cell density as the mixes. We placed the filters in Petri dishes atop a single filter pad (Pall), which was soaked in 1.5 ml of PDF buffer (20.1 mM KCl, 5.3 mM MgCl_2_·6H_2_O, 9.2 mM K_2_HPO_4_, 13.2 mM KH_2_PO_4_, 0.5 g/l streptomycin sulfate, pH = 6.4), placed them in a humid chamber, and incubated them at 22 °C in the dark for development. Following fruiting body formation (∼24 h), we picked at least ten individual fruiting bodies randomly from each mix filter. We resuspended the spores from each fruiting body in 10 μl detergent to eliminate amoebae and counted the spores using phase contrast and fluorescent microscopy to determine the proportion of GFP-positive spores. We counted approximately 400 spores for each fruiting body.

### Cell tracker staining.

We grew each wild isolate to mid-exponential phase in association with K. pneumoniae on SM-agar plates, washed the cells twice in cold KK2 buffer, and resuspended them at a density of 1 × 10^7^ cells/ml. We stained the cells with CellTracker Green CMFDA (Molecular Probes) according to the manufacturer's recommended protocol with the following modifications. We added the cell tracker reagent at a concentration of 50 μM, incubated the cells for 30 min, washed them twice with cold KK2 buffer, and incubated them for another 30 min in KK2 buffer to allow the cells to efflux the excess dye. Following staining, we resuspended the cells at a density of 6 × 10^7^ cells/ml, mixed them in equal proportions with unlabeled cells, and deposited the mix on filters, as described above. Following fruiting body formation, we harvested the spores from individual fruiting bodies in detergent and analyzed the proportion of fluorescent spores on a BD LSRII flow cytometer.

### Microsatellite genotyping.

We genotyped the strains at 12 microsatellite loci, which were dispersed throughout the genome. These microsatellite loci were designed based on the AX4 sequence (our focal strain) and described previously [47]. To extract genomic DNA, we collected spores from 5–10 fruiting bodies and incubated them in a mixture of 150 μl of 5% Bio-Rad Chelex-10 and 10 μl of 20 mg/ml proteinase K for 4 h at 56 °C, followed by 30 min at 98 °C. Each microsatellite locus was amplified by PCR using fluorescently labeled primers (Table S2). The resulting product was analyzed on an ABI 3100 sequencer, and the programs GeneScan 3.7 and GENOTYPER were used to determine the fragment size. To estimate the genetic distances, we calculated the standardized Euclidean distance between strains, using the PCR product size as a quantitative variable (Table S1). Relatedness and genetic similarity based on multi-locus, multivariate clustering techniques incorporating Euclidean distances between haplotypes have been described previously [48–51]. We used a standardized Euclidean distance metric, which scales each locus by its variance, such that they contribute equally [52]. Clustering based on allele size rather than number of shared alleles offers greater resolution of genetic differences, particularly for more distantly related individuals. It is analogous to a stepwise mutational model (SMM). SMM have been shown to perform well [53–56], particularly when microsatellite mutation rates are low and there is not a strong directional bias in the allele length changes, both of which have been shown for these loci (described in [47]). Analyses were repeated where distances were calculated based on the presence of shared alleles, where alleles were considered identical if the estimated size was within 3 bp of the allele for AX4.

### Strain construction.

We inoculated spores of natural isolate QS44 from fruiting bodies into Petri dishes containing HL5 medium supplemented with 10% fetal bovine serum and grew the resulting cells in submerged culture until they fully covered the surface. We transformed the cells with an expression vector containing the S65T-GFP reporter driven by the act15 promoter. Transformation conditions were modified from protocols described previously [57]. We harvested the cells after washing twice in ice-cold KK2 buffer and resuspended them at 1 × 10^8^ cells/ml in ice-cold H-50 buffer. We mixed 100 μl of cell suspension with 5–15 μg of plasmid in a 0.1-cm-gap electroporation cuvette and electroporated the mixture at 0.95 KV and 25 mF three times at approximately 5-s intervals. The transformants were grown in submerged culture with HL5 supplemented with 8 μg/ml G418, and selected clones were tested for GFP-expression using fluorescent microscopy. We verified the genetic background using microsatellite genotyping.

### Statistical methods.

Proportions were arcsine square root transformed to ensure that the variance was statistically independent of the mean, and thus to account for differences among mixes in the overall proportion of fluorescent spores [58]. The transformation does not affect the statistical significance of the results. The genetic distances were non-normally distributed, so we also performed a nonparametric (Spearman's rank) correlation. All pairwise mixes against the reference strain AX4-GFP were performed a minimum of three times and used to calculate an average variance. Three of the mixes (NC4, QS43, and QS45) were replicated four times. In such cases, we computed the average variance across all four replicates, although the inclusion of the fourth replicate did not affect the statistical significance of our results.

## Supporting Information

Table S1 *Dictyostelium* Strains Used in This Study (82 KB DOC)

Table S2 PCR Primers for Amplification of 12 Microsatellite Loci (58 KB DOC)

## Abbreviations

GFP: green fluorescent protein

## Note Added in Proof

Similar findings have been reported in Dictyostelium purpureum by Mehdiabadi et al. [59].
