# Supplementary material for: Kin Discrimination Increases with Genetic Distance in a Social Amoeba
Source: PLoS Biol. 2008 Nov 25;6(11):e287. doi: 10.1371/journal.pbio.0060287 (PMC2586364; doi:10.1371/journal.pbio.0060287)
Supplement: Table S1 — (82 KB DOC) [file pbio.0060287.st001.doc]

Supplementary Material

Table S1. *Dictyostelium* strains used in this study

| Strain | Location Isolated | GPS coordinates | Microsatellite fragment size (bp) | | | | | | | | | | | | Genetic distance to reference strain AX4^a^ |
| --- | --- | --- | --- | --- | --- | --- | --- | --- | --- | --- | --- | --- | --- | --- | --- |
|  |  |  |  |  |  |  |  |  |  |  |  |  |  |  |  |
|  |  |  | L307 | L308 | L317 | L319 | L323 | L327 | L328 | L329 | L330 | L345 | L357 | L366 |  |
| AX4^b^ | Little Butts Gap, NC | 35°46.317’ N 82°20.533’ W | 236 | 236 | 192 | 385 | 441 | 230 | 243 | 248 | 215 | 245 | 250 | 243 | 0 |
| NC4 | Little Butts Gap, NC | 35°46.317 N 82°20.533’ W | 236 | 236 | 192 | 385 | 438 | 230 | 243 | 248 | 215 | 245 | 250 | 240 | 0.13 |
| QS32 | Pasadena, TX | 29°35’ N  95°4’ W | 184 | 133 | 202 | 379 | 438 | 108 | 188 | 234 | 143 | 234 | 269 | 206 | 5.16 |
| QS33 | Webster, TX | 29°32’ N  95° 9’ W | 184 | 133 | 202 | 379 | 438 | 108 | 188 | 234 | 143 | 234 | 269 | 206 | 5.16 |
| QS34 | Bloomington, IN | 39˚13.227' N  86˚21.534' W | 159 | 133 | 192 | 326 | 389 | 197 | 258 | 185 | 159 | 233 | 225 | 209 | 4.61 |
| QS113 | Effingham, IL | 39°5.467’ N 88°34.833’ W | 184 | 133 | 204 | 289 | 390 | 181 | 185 | 215 | 186 | 239 | 266 | 240 | 4.27 |
| QS36 | Land Btw Lakes, KY | 36°59.856’ N 88˚13.132' W | 150 | 151 | 168 | 254 | 343 | 169 | 185 | 157 | 127 | 234 | 238 | 230 | 6.17 |
| QS37 | Linden, TX | 33°3.710' N 94°16.414’ W | 129 | 198 | 143 | 233 | 346 | 168 | 191 | 157 | 162 | 230 | 296 | 274 | 6.39 |
| QS38 | Mt. Lake, VA | 37°21’ N  80° 31’ W | 160 | 136 | 161 | 328 | 386 | 197 | 191 | 246 | 189 | 233 | 256 | 209 | 4.46 |
| QS39 | Indian Gap, TN | 35°36.606' N 83°26.821' W | 159 | 139 | 195 | 251 | 434 | 221 | 218 | 153 | 215 | 239 | 250 | 270 | 4.71 |
| QS40 | Mt.Greylock, MA | 42°38.200’ N 73°10.367’ W | 205 | 142 | 183 | 263 | 365 | 157 | 247 | 221 | 119 | 195 | 266 | 265 | 5.02 |
| QS41 | Little Butts Gap, NC | 35°46’ N 82°20’ W | 197 | 195 | 155 | 251 | 429 | 221 | 247 | 153 | 215 | 233 | 250 | 191 | 4.23 |
| QS42 | Monteverde, Costa Rica | 10°18' N 84°26' W'^c^ | 131 | 101 | 266 | 246 | 330 | 193 | 177 | 158 | 136 | 104 | 173 | 251 | 8.46 |
| QS43 | La Malintzi Park, Mexico | 19°12.850’ N 98°6.467’ W | 166 | 254 | 173 | 199 | 273 | 130 | 233 | 166 | 149 | 296 | 177 | 203 | 6.91 |
| QS44 | Mt. Fuji, Japan | 35°25’ N 138°41’ E | 190 | 133 | 202 | 379 | 438 | 108 | 188 | 234 | 143 | 234 | 263 | 207 | 5.07 |
| QS6 | Mt. Lake, VA | 37°21’ N  80° 31’ W | 163 | 136 | 162 | 339 | 387 | 181 | 224 | 249 | 162 | 233 | 217 | 209 | 4.45 |

^a^Genetic distance between strains was estimated as the standardized Euclidean distance based on PCR fragment size (which is proportional to the number of repeats) at 12 microsatellite loci that were distributed throughout the genome. See Methods for more detail.

^b^AX4 is a derivative of the axenic strain AX3, which was derived from the wild isolate NC4 37 years ago[1].

^c^GPS coordinates for this isolate approximated using Google Earth.

References

1. Loomis WF (1971) Sensitivity o*f Dictyostelium discoide*um to nucleic acid analogues. Experimental Cell Research 64: 484-486.
