# Supplementary material for: Kin Discrimination Increases with Genetic Distance in a Social Amoeba
Source: PLoS Biol. 2008 Nov 25;6(11):e287. doi: 10.1371/journal.pbio.0060287 (PMC2586364; doi:10.1371/journal.pbio.0060287)
Supplement: Table S2 — (58 KB DOC) [file pbio.0060287.st002.doc]

Table S2. PCR primers for amplification of 12 microsatellite loci

| Locus | Direction | Primer Sequence (5’ to 3’ direction) |
| --- | --- | --- |
| 307 | Forward | AGGATAGCTCTCAGCCATCAA |
|  | Reverse^1^ | AATGTTGGTTGGGATGATGA |
| 308 | Forward^1^ | CCTGAACAAACACATTCCTCAA |
|  | Reverse | GGGGTTATTGTTGGTGCTGA |
| 317 | Forward^1^ | CAATACCACCACCACCACAG |
|  | Reverse | GGTGGCGATGATGATGTAGTT |
| 319 | Forward | GAGTCGATGTAATCAACCATCAG |
|  | Reverse^1^ | AAAACTGGTACTGCAACCACAA |
| 323 | Forward | TTGGAAAAAGCCAACAACCT |
|  | Reverse^1^ | TCAAAGTCCATGGTACAAAACC |
| 327 | Forward | TGGACAACAACCAATTCAACA |
|  | Reverse^1^ | TGTGGCTGAAAATTAGGGTCA |
| 328 | Forward^2^ | TTGATCAAAAGATACATCATTATTTGG |
|  | Reverse | TGATCAACAGCAACAACAACAA |
| 329 | Forward | CACAAACCTCAACTTCAACAACA |
|  | Reverse^2^ | TTGGTTTTGTTGATGACTCAA |
| 330 | Forward^1^ | TTAATCAAAGTCAAATTGGTTTACAA |
|  | Reverse | ATTATTGTTATTTGATGATGATGATGT |
| 345 | Forward^1^ | TGTTAAGCCACAACCAA |
|  | Reverse | TTTGTTGGTTGTTGTTGTTCG |
| 357 | Forward^1^ | CAATTGGTGAATTTGCTCTAATTT |
|  | Reverse | AAAGAAGAAGAGATTGGTAATCAAGA |
| 366 | Forward | TCAAATCAACTTTGGGAGCA |
|  | Reverse^2^ | TTTGTTGGT TGTTGTTGTTGC |

^1^Fluorescently labeled with 5’ HEX (green).

^2^Fluorescently labeled with 5’ 6-FAM (blue).
